# Comparison of Hormone-Sensitive Oligorecurrent Prostate Cancer Patients Based on Routine Use of Choline and/or PSMA PET/CT to Guide Metastasis-Directed Therapy

**DOI:** 10.3390/cancers15061898

**Published:** 2023-03-22

**Authors:** Raphaël Metz, Aurore Rauscher, Loïg Vaugier, Stéphane Supiot, Franck Drouet, Loic Campion, Caroline Rousseau

**Affiliations:** 1 Nuclear Medicine Department, Institut de Cancérologie de l’Ouest, Boulevard J. Monod, F-44800 St-Herblain, Francecaroline.rousseau@ico.unicancer.fr (C.R.); 2 Radiation Oncology Department, Institut de Cancérologie de l’Ouest, Boulevard J. Monod, F-44800 St-Herblain, France; 3 Laboratoire US2B, Unité en Sciences Biologiques et Biotechnologies, UMR CNRS 6286, UFR SCIENCES ET TECHNIQUES, 2, Rue de la Houssinière, F-44322 Nantes, France; 4 Radiation Oncology Department, Clinique Mutualiste de l’Estuaire, F-44600 Saint-Nazaire, France; 5 Biostatistics Department, Institut de Cancérologie de l’Ouest, Boulevard J. Monod, F-44800 St-Herblain, France; 6 Nantes Université, Univ Angers, INSERM, CNRS, CRCI2NA, F-44000 Nantes, France

**Keywords:** prostate cancer, oligorecurrent, PET/CT, [68Ga]Ga-PSMA, [18F]F-choline, external radiotherapy

## Abstract

**Simple Summary:**

Advances in metabolic imaging with 18F-choline (FCH) and, more recently, 68Ga-PSMA PET/CT have revolutionized the biochemical recurrence (BR) prostate cancer imaging assessment and allowed the emergence of the oligometastatic disease concept. Several phase II studies suggested that oligometastases local treatment with external beam radiation therapy (EBRT) improved biochemical recurrence-free survival (BR-FS). The diagnostic performance of 68Ga-PSMA PET/CT is superior to FCH for BR when the PSA value is low. The aim of our retrospective study was to evaluate the clinical impact of PET/CT on two groups of patients with oligorecurrence and PSA < 2 ng/mL treated locally by EBRT guided by 68Ga-PSMA or FCH PET/CT. In 123 patients with a median follow-up of 42.2 months, we showed that 68Ga-PSMA PET/CT-guided EBRT appeared to improve BR-FS compared to FCH PET/CT-guided EBRT. PSA doubling time at recurrence appeared to be a prognostic factor associated with treatment response.

**Abstract:**

Background: In hormone-sensitive oligorecurrent prostate cancer (PC), the literature showed [68Ga]Ga-PSMA (PSMA) and [18F]F-choline (FCH) PET/CT can successfully guide metastasis-directed therapies (MDT). This observational retrospective study aimed to explore, in routine use, the impact of FCH or PSMA PET/CT in guiding MDT for hormone-sensitive oligometastatic PC at different recurrences. Methods: In 2017–2020, patients initially treated with radical prostatectomy but, in biochemical recurrence (with PSA ≤ 2 ng/mL), diagnosed as oligometastatic based on FCH or PSMA PET/CT, were identified. MDT was stereotactic body radiotherapy (SBRT), elective nodal or prostate bed radiotherapy ± boost and ± androgen deprivation therapy (ADT). The primary endpoint was biochemical relapse-free survival (BR-FS), defined as a PSA increase ≥ 0.2 ng/mL above the nadir and increasing over two successive samples and the secondaries were ADT-free survival (ADT-FS). Results: 123 patients (70 PSMA and 53 FCH) were included. The median follow-up was 42.2 months. The median BR-FS was 24.7 months in the PSMA group versus 13.0 months in the FCH group (*p* = 0.008). Similarly, ADT-FS (*p* = 0.001) was longer in patients in the PSMA group. In multivariate analysis, a short PSA doubling time before imaging (*p* = 0.005) and MDT with SBRT (*p* = 0.001) were poor prognostic factors for BR-FS. Conclusions: Routine use of FCH or PSMA PET/CT in hormone-sensitive PC showed an advantage for using PSMA PET/CT to guide MDT in terms of BR-FS and ADT-FS in patients with low PSA value. Prospective studies are needed to confirm these hypotheses.

## 1. Introduction

Among prostate cancer (PC) patients treated radically (radical prostatectomy RP or prostatic external radiotherapy RT), 27 to 53% will develop a biological recurrence (BR) [1]. BR after RP is defined as a rise in prostate-specific antigen (PSA) ≥ 0.2 ng/mL confirmed by two successive samples [2]. Recurrent patients thus form a heterogeneous group with different therapeutic responses and prognostic profiles. The European Association of Urology (EAU) recently established a stratification between “EAU low-risk” BR (PSA doubling time (PSA DT) > 1 year and/or ISUP score < 4) and “EAU high-risk” (PSA DT < 1 year and/or ISUP score ≥ 4) [3]. 

In the case of BR, it is crucial to determine whether the disease remains (confined to the pelvis or is already more remote) to adopt the therapeutic strategy: locoregional treatment targeted by RT, systemic treatment by androgen deprivation treatment (ADT), or a combination of both. The time between the recurrence and the initial treatment, PSA DT, and initial ISUP score have a predictive value of the recurrence site: local or metastatic [4]. For a long time, these criteria were the only ones available. They used to decide the therapeutic choice, probably reflecting a need not met with conventional imaging that was nevertheless performed. Conventional imaging techniques (computed tomography and bone single photon emission computed tomography) are sub-optimal for identifying early recurrences after radical treatment [5,6]. Progress in molecular imaging and the emergence of new, more sensitive, positron emission tomography (PET) radiopharmaceuticals have revolutionized BR imaging. The first radiopharmaceutical available, [18F]F-choline PET/CT (FCH-PET), presented in BR with interesting sensitivities and specificities evaluated at 85–96% and 73–97%, respectively according to the recent meta-analysis by Wang et al. in 2021 [7]. However, its performance is strongly influenced by PSA value and kinetics at the time of imaging. The sensitivity of FCH-PET drops when the PSA is lower than 2 ng/mL and/or the PSA DT > 6 months [8,9,10,11]. This is why FCH-PET is not recommended for BR assessment if the PSA is lower than 1 ng/mL, according to the French Association of Urology [2]. New radiopharmaceuticals have emerged for BR imaging assessment: prostate-specific membrane antigen (PSMA) ligands. Many studies show their higher performances compared to FCH-PET, especially with low PSA values [12,13,14,15,16]. According to the meta-analysis by Perera et al., BR detection rates were 33%, 45%, 59%, 75% and 95% in cases of PSA lower than 0.2 ng/mL or between [0.2–0.5], [0.5–1], [1–2], and higher than 2 ng/mL respectively [17]. These performances have led to interesting changes in BR therapeutic management in many cases [18,19,20,21]. As a result, the European Association of Nuclear Medicine recommends performing [68Ga]Ga-PSMA PET/CT (PSMA-PET) as the first-line for BR detection, even with a very low PSA value (≤1 ng/mL) where FCH-PET is not indicated [22].

Progress in molecular imaging, making possible more precise and early localization of recurrent disease, has led to the emergence of a new concept: oligorecurrent PC, an intermediate state between localized disease and extensive metastatic disease [23]. The definition of oligometastatic disease is not unequivocal and varies according to the studies, generally ranging from 3 to 5 metastatic sites. It also depends on the imaging techniques used, making it possible to detect the recurrence more or less precisely. In some cases, the oligometastatic stage is only the tip of the iceberg of subclinical polymetastatic disease [24]. 

Concomitantly with the emergence of innovative PET imaging techniques, new therapeutic possibilities have been developed: RT-targeted treatment of recurrence, interventional radiology, or even salvage surgery. Without sufficient data, there are no precise recommendations for managing PC oligometastatic relapses. Several phase II multicentre randomized trials suggest that treating oligometastases directly with stereotactic body radiotherapy (SBRT) improves disease-free survival [25,26,27,28] and delays the introduction of ADT [29,30], thereby improving patients’ quality of life. In the ORIOLE phase II study, Phillips et al. randomized 54 patients with oligometastatic recurrence (3 or fewer lesions seen on conventional imaging) after RP into two groups. The 1st group benefited from metastasis SBRT and the 2nd from surveillance. SBRT statistically reduced the proportion of patients progressing at 6 months: 19% vs. 61% (*p* = 0.005). The median progression-free survival was not reached at 18 months. It was 5.6 months in the surveillance group (*p* = 0.002). All the patients treated with SBRT had also had a PSMA-PET, the result of which was not used for SBRT but which found additional metastases in 44.4% (16/36) of the patients, highlighting the quality differences in the staging assessment depending on the imaging used, something that must be kept in mind. The proportion of patients with progression at 6 months was 38% in patients with untreated lesions on seen PSMA-PET versus 5% in those without untreated lesions (*p* = 0.03). Patients with all PSMA-PET lesions treated also had median progression-free survival (*p* = 0.006) and longer distant metastasis-free survival (*p* < 0.001) [27]. More sensitive PSMA-PET would make it possible to select patients better and to guide the targeted RT metastasis treatment more optimally.

Therefore, it appears essential to use the best radiopharmaceuticals available to detect recurrence as exhaustively as possible, plan treatment with RT optimally, and thus improve therapeutic efficacy. As previously described, PSMA-PET performed at BR has better detection rates than FCH-PET at low PSA values (≤2 ng/mL). For this reason, it seemed interesting to us to compare the efficacy of RT treatment guided by PSMA-PET or FCH-PET in BR patients with low PSA values. The literature reports 3 retrospective studies comparing RT treatment guided by PSMA-PET or FCH-PET on small cohorts of BR patients with heterogeneous PSA values between the groups [31,32,33]. Two of these suggest that PSMA-PET-guided SBRT obtained better results than FCH-PET [31,32]. On the other hand, the other study did not find any difference in biological recurrence-free survival (BR-FS) between the two groups [33]. The debate remains open.

This study aims to evaluate the clinical impact of PSMA-PET and FCH-PET on two groups of PC patients in BR with a PSA value ≤ 2 ng/mL imaged by either one of these radiopharmaceuticals, then treated with image-guided RT on the local or oligometastatic PC lesions detected. 

## 2. Materials and Methods

We present a single-centre observational retrospective study carried out in accordance with the principles of the Declaration of Helsinki. It was approved by the Ethics Committee of the University Hospital (2022-018). All patients were informed of the use of their clinical data for research purposes, and none of them refused this use.

### 2.1. Population and Data

Inclusion criteria were: (1) patients with histologically-proven PC initially treated with RP, (2) patients with hormone-sensitive PC in BR proven by a rising PSA ≥ 0.1 ng/mL and no more than PSA ≤ 2 ng/mL, (3) patients who had FCH or PSMA-PET and in whom at least one positive lesion whatever the localization (local, node, bone or visceral) has been detected with a maximum of 5 lesions per patient, (4) patients who then received radiotherapy treatment for all lesions seen on PET/CT regardless of the radiopharmaceutical.

All patients who underwent FCH or PSMA-PET from September 2017 to May 2020 and met the inclusion criteria were included. In addition, most patients who underwent a PSMA-PET were also included in the protocol (NCT03443609), which provided a PSMA-PET alone. The other had PMSA-PET, thanks to a temporary authorization to use it if negative FCH-PET was first observed.

Biochemical recurrence-free survival (BR-FS) was the primary endpoint of the study, defined as the time between the end of RT and rising PSA ≥ 0.2 ng/mL above the nadir and increasing over two successive samples. ADT-free survival (ADT-FS) was analyzed and defined as the time from the end of metastasis-directed therapies (MDT) to the introduction of continuous ADT. Evolution in PSA values (PSA response) at 3–6 months from the end of RT was assessed only in patients who had not received concomitant ADT. The PSA test was performed a few days before RT. As described in the literature, the response was defined as complete if PSA decreased by at least 50% compared to pre-RT PSA, partial if PSA decreased from 10% to 50%, stable if PSA values moved by ±10%, and progressing if PSA increased by more than 10% [34,35,36]. Potential RT adverse events and acute toxicities were monitored and assessed using the CTCAE v5.0 (Common Terminology Criteria for Adverse Events).

### 2.2. Radiopharmaceuticals and PET/CT Acquisition

PSMA was synthesized in the Nuclear Medicine unit radiopharmacy. [^68^Ga]Ga-PSMA-HBEDCC (PSMA) was prepared using a procedure similar to that described by Eder et al. [37] transferred to a cassette-based automated synthesis module (Modular-Lab, PharmTracer; Eckert & Ziegler, Berlin, Germany) based on acetone-free cation exchange post-processing. All imaging was performed on a PET/CT Biograph mCT and Vision 450 (Siemens, Erlangen, Germany). Images were acquired (3 min per step) 1 h after PSMA injection with an activity of 2 MBq/kg and 30 min after FCH injection of 3 MBq/kg, from the vertex to the root of the thighs, with the arms above the head. Lasilix^®^ was administered, if possible, during both types of PET/CT. Low-dose CT 3D acquisition without contrast product injection was performed: auto mA according to the patient’s weight; 80–140 kV; 3 mm slice thickness. Images were reconstructed with Ultra HD and TOF: 3 iterations, 21 subsets with a Gaussien 3D filter (FWHM 2 mm). Following recently published guidelines [38], any PSMA foci above the background level, which could not be explained by physiological uptake or other known pitfalls, were considered PSMA-positive.

### 2.3. Treatments and Follow-Up

RT was planned by each patient’s referring radio-oncologist, knowing the results of the PSMA or FCH-PET imaging. This treatment was carried out in two regional centres in a similar way. RT techniques did not depend on the choice of radiopharmaceutical used. In the indication of elective node irradiation with an additional dose at the node oligometastasis, intensity-modulated radiotherapy (IMRT) delivered 54 Gy in 30 fractions of 1.8 Gy on the elective pelvic node volume with a simultaneous integrated boost of 66 Gy in 30 fractions of 2.2 Gy on the nodes deemed positive by PET/CT [28]. The elective pelvic node volume was determined according to the international recommendations of the RTOG [39] GETUG [40]. Patients who had not received prior irradiation on the prostate bed received 66 Gy in 2 Gy fractions in this area [41]. In the indication for nodal SBRT, this was performed using the dynamic conformational arc (DCA) technique and delivered 29.7 or 33 Gy (80% isodose) in 3 fractions at the periphery of the planned tumor volume (PTV). For relapse of previously irradiated prostate bed, reirradiation under stereotaxic conditions made it possible to deliver 30–36 Gy in 5 or 6 fractions of 6 Gy using the IMRT technique on the lesion visualized by PET/CT. Bone lesion SBRT delivered 27–35 Gy (80% isodose) in 3–5 fractions to the periphery of the PTV. Treated patients could receive injections of LHRH (luteinizing hormone-releasing hormone) agonists or antagonists intermittently (for a maximum duration of 6 months or 2 years). Clinical surveillance and biological monitoring of PSA were generally carried out at 6 weeks and 3 months, then every 6 months.

### 2.4. Statistical Analyzes

The first step was an overall descriptive analysis. The qualitative factors were described by the frequency of their respective modalities and the continuous factors by their mean ± standard deviation (or median range). BR-FS (primary endpoint) and ADT-FS were described using Kaplan-Meier curves in the overall population. The population monitoring median was calculated with the inverse Kaplan-Meier method.

Due to the study’s design and the small number of patients, the matched-pair analysis was impossible. Therefore, the second step was comparative. The two subgroups (FCH and PSMA-PET) were compared using the:

- Pearson’s chi-square test (or Fisher’s test if necessary) for qualitative variables.
- Student’s *t* test (or Mann-Whitney test if necessary) for continuous variables.
- The Log-Rank test for survival data (BR-FS, ADT-FS); Kaplan-Meier curves were plotted.

At the same time, the univariable impact on the BR-FS of each of the other possible confounding factors was determined using the Log-Rank test for the qualitative factors and the univariable Cox model for the continuous factors.

The third stage was multivariable. Confounding factors were considered to assess the independent prognostic impact of the main factor (FCH and PSMA-PET) on BRFS. To maximize the robustness of the results and to reduce selection and confounding biases caused by unbalanced prognostic factors between the groups (FCH and PSMA-PET), a multivariable analysis using the semi-parametric Cox model made it possible to calculate the adjusted hazard ratio [HR] of relapse treatment (FCH and PSMA-PET) with a 95% confidence interval (CI). The variables significant at the 10% univariable level and the known prognostic variables were introduced into the Cox multivariable model, and the proportional hazards hypothesis of the final model was verified. All analyzes were performed with a final significance level set at 5% (two-sided formulation) using SAS software, version 9.4 (SAS Institute Inc., Cary, NC, USA) and Stata SE 17 (StataCorp LLC, College Station, TX, USA). 

## 3. Results

### 3.1. Population, Lesions, and Treatment Characteristics

All characteristics are detailed respectively in Table 1, Table 2 and Table 3. Analysis of Table 1 showed that 123 patients with local or oligometastatic recurrence were included: 70 in the PSMA-PET group and 53 in the FCH-PET group. Of the patients in the PSMA-PET group, 18 (25.7%) had previously negative FCH PET/CT. All the initial PC characteristics were homogeneous between the two groups, the PSA DT before imaging and the EAU classification risk group at the BR. On the other hand, the mean PSA value before imaging was significantly higher in the FCH-PET group than in the PSMA-PET group (*p* < 0.001). In addition, patients in the FCH-PET group had more frequently received prostate bed RT with elective pelvic node RT and ADT before PET/CT imaging, compared to the PSMA-PET group (*p* < 0.010). Thus, patients in the PSMA-PET group were more frequently naïve to any treatment complementary to RP than those in the FCH-PET group (*p* = 0.014). 

Table 2 reports all lesion sites, i.e., a total of 204 lesions: 112 in the PSMA-PET group and 92 in the PET-FCH group. The lesion sites detected by PET/CT were in order of frequency: pelvic lymph nodes (111/204, 54.4%), bone (35/204, 17.2%), prostate bed (34/204, 16.7%), extra-pelvic lymph nodes (21/204, 10.3%), and visceral (3/204, 1.4%). Regarding bone locations, they were mainly situated on the spine, pelvis, or sacrum (28/35; 80.0%), then on the ribs (5; 14.3%), and finally on the peripheral skeleton (2; 5.7%). Nineteen patients had a prostate bed recurrence only, 12 (17.1%) in the PSMA-PET group and 7 (13.2%) in the FCH-PET group. No significant difference was observed between the two groups in terms of the number of lesions or their location.

Table 3 shows the different RT treatments decided and guided by PET/CT imaging. Patients in the PSMA-PET group benefited more frequently from elective pelvic node RT with boost (42.9% versus 17.0% for the FCH-PET group; *p* = 0.002). Conversely, for patients in the PET-FCH group, it was more frequently decided to perform SBRT on node and bone lesions (11.3% versus 1.4% for the PSMA-PET group; *p* = 0.042). Concomitant intermittent ADT was instituted in 34.0% of patients in the FCH-PET group versus 18.6% in the PSMA-PET group (*p* = 0.028).

### 3.2. Study of the Different Survivals after Treatment

**Figure 1:**
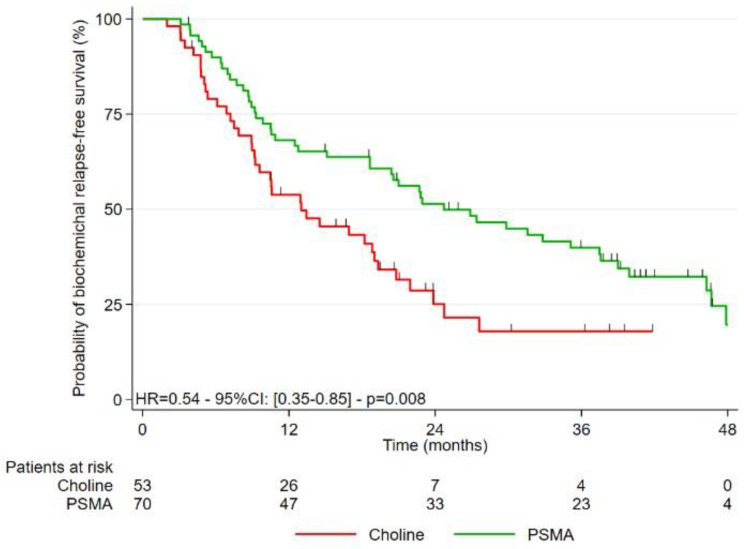
Kaplan-Meier curves for biochemical recurrence-free survival (BR-FS).

**Figure 2:**
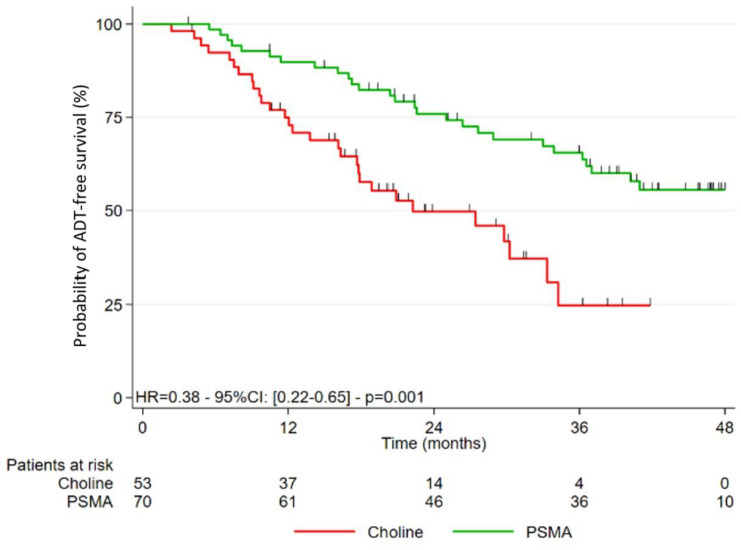
Kaplan-Meier survival curve without ADT (ADT-FS).

**Figure 3:**
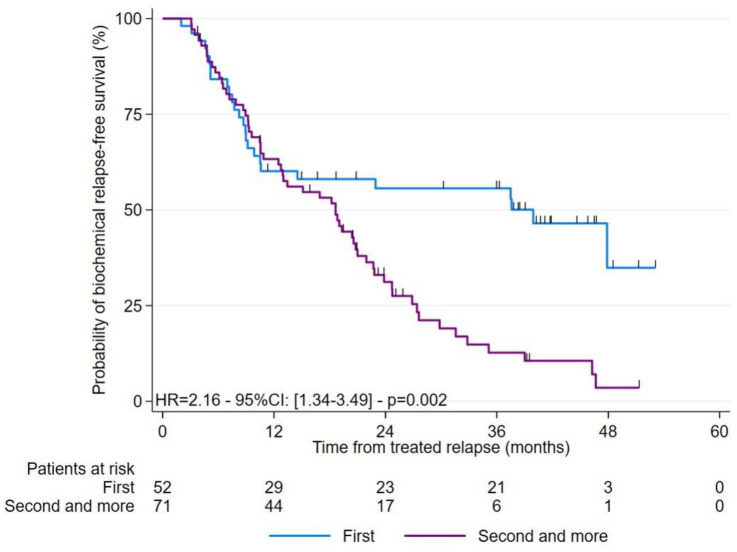
Kaplan-Meier curves for BR-FS according to patients with their 1st or (2nd–3rd–4th) BR before RT guided by PSMA or FCH PET/CT.

The median follow-up for the entire cohort was 42.2 months (95%CI: 36.5–45.8). The different types of survivals analyzed are presented in Figure 1, Figure 2 and Figure 3. BR-FS was significantly longer in the PSMA-PET group, with a median of 24.7 months (95%CI: 18.6–37.5) versus 13.0 months (95%CI: 9.2–19.0) in the FCH-PET group (*p* = 0.008). 

Patients in the PSMA-PET group also had longer ADT-FS than patients in the FCH-PET group (*p* = 0.001). ADT-FS rates at 2 years and 3 years were 75.9% (95%CI: 63.7–84.5) and 65.5% (95%CI: 52.3–75.8) compared to 49.7% (95%CI: 34.5–63.3) and 24.7% (95%CI: 9.6–43.4), respectively in the PSMA-PET and FCH-PET groups (*p* = 0.001).

Our cohort included patients with their 1st BR and patients with more recurrent disease in their 2nd, 3rd and 4th BR. Patients with a 2nd relapse or more presented a shorter BR-FS than patients with a 1st relapse, regardless of the radiopharmaceutical used (HR = 2.16; 95%CI: 1.34–3.49; *p* = 0.002).

The sub-group analysis, including only patients with a 2nd or more recurrence, highlighted a trend towards a longer median BR-FS for the PSMA-PET group than for the FCH-PET group (20.6 versus 13.4 months; *p* = 0.085). Similarly, ADT-FS tended to be longer in the PSMA-PET group than the FCH-PET group (36.2 versus 29.8 months; *p* = 0.060).

### 3.3. Study of the PSA Response at 3–6 Months Post-RT

Analysis of Table 4 shows that a majority of patients achieved a PSA response at 3–6 months post-RT (*n* = 60/95, 63.1%), and no difference was observed between the PSMA-PET (*n* = 37/57, 64.9%) and FCH-PET (*n* = 23/38, 60.5%) groups (*p* = 0.912). 

According to Table 5, a significantly greater proportion of PSA responses at 3–6 months post-RT was obtained in patients who received irradiation in all pelvic or extra-pelvic node areas (*p* < 0.001). On the other hand, after bone lesions or pelvic node SBRT, a poorer PSA response at 3–6 months post-RT was obtained (*p* = 0.031 and *p* = 0.044).

In terms of survival, patients who obtained a PSA response at 3–6 months post-RT had better ADT-FS (HR = 0.23; 95%CI: 0.13–0.40; *p* < 0.001) than those who did not obtain a response.

### 3.4. Analysis of Predictive Factors Related to BR-FS

The results of univariate and multivariate Cox regression analyses looking for independent predictors of BRFS are detailed in Table 6.

In univariate analysis, none of the initial PC variables (diagnosis PSA value, surgical status, and ISUP) was significantly related to BR-FS. 

On the multivariate analysis, including all the significant data from the univariate analysis, it appears that only two of them remain independent prognostic factors for BR-FS: a long PSA DT at the time of the PET/CT had a better prognosis (HR = 0.94; 95%CI: 0.91–0.98; *p* = 0.005), and benefiting from RT on the prostate bed, associated or not with elective pelvic node RT instead of oligometastasis SBRT, was favorable (HR = 0.33; 95%CI: 0.18–0.61; *p* = 0.001). However, after adjusting for the two previous variables, the diagnostic modality that guided the RT (FCH or PSMA-PET) was not predictive (HR = 0.97; 95%CI: 0.56–1.70; *p* = 0.924).

## 4. Discussion

To our knowledge, 3 major points in our study need to be underlined and allow it to stand out in the literature, namely, the 3 studies comparing the efficacy of treatment focused on oligo-recurrences using RT guided by FCH or PSMA in clinical routine for PC recurrence [31,32,33]. The main results of these studies are detailed in Table S1.

First, to our knowledge, our study is the first to compare the efficacy of RT guided by PSMA or FCH PET/CT only in patients with local or oligometastatic recurrence with PSA values ≤ 2 ng/mL. This gives our population homogeneity regarding both radiopharmaceuticals. The value of this threshold choice was twofold: it seemed to be adapted to early oligometastatic recurrences and, therefore, to focal treatments such as RT, but also, PSA values ≤ 2 ng/mL correspond to differences in the diagnostic performances of the 2 radiopharmaceuticals [12].

On the other hand, this study, thanks to a median follow-up higher than that found in the literature [31,32,33], i.e., 42.2 months, demonstrated the prolonged efficacy of treatment using RT guided by PET/CT on both BR-FS and ADT-FS in the PET-PSMA group. The differences in duration of BR-FS and ADT-FS between the two groups could be explained by the more exhaustive PSMA PET/CT assessment when the PSA is ≤2 ng/mL due to the known performances of the two radiopharmaceuticals [8,9,10,11,12,13,14,15,16]. Thus, the recurrence assessment imaging with PSMA PET/CT may reflect the reality of the tumor burden more faithfully and may make it possible to select patients who “really” have an oligometastatic recurrence and for whom targeted treatment guided by the image is beneficial. In addition, the good sensitivity of PSMA PET/CT at low PSA values makes it possible to detect recurrences earlier and perform RT earlier, which may impact therapeutic efficacy. Our results in ADT-FS are consistent with those of 2 other studies by Mazzola et al. [31] and Deijen et al. [32]. ADT-FS reflects survival with an appreciable quality of life. Postponing the introduction of long-term ADT is a real clinical advantage for patients, delaying the onset of side effects of ADT, such as increased cardiovascular risk [42], the onset of a metabolic syndrome, osteoporosis, psychic disorders, or even sexual disorders, altering quality of life [43]. The 2 studies, for their part, were not interested in BR-FS. However, we have shown that the BR-FS rates at 2 and 3 years were significantly higher for the PET-PSMA group than the PET-FCH group (*p* = 0.008). Although BR is not a clinical criterion, BR-FS is important to consider because it quantifies the real effectiveness of the treatment applied to the patient. However, our results differ from those of Schmidt-Hegemann et al., where no difference was found in BR-FS between the two groups of patients [33]. In their study, these authors’ patients with persistent PSA following PR or in a first BR were included with or without lesions detected by PET/CT. Schedules for RT treatment received were not, for some patients (41.9% had a negative PET/CT), guided by at least one lesion detected by PET/CT, but chosen according to a probabilistic event based on clinical patient data. This may explain the results of this study. 

Finally, to highlight the prognostic factors impacting BR-FS, this study found two negative factors for BR-FS in multivariate analysis. First, a short PSA DT before PET/CT imaging with an HR of 0.94 (95%CI 0.91–0.98; *p* = 0.005). Although the HR is close to 1, the PSA DT at relapse is significantly associated with a poor prognosis, consistent with the literature data [44]. Two other studies [32,33] found PSA absolute values to be a factor for poor prognosis in multivariate. However, these studies presented a much wider distribution of PSA values than that observed in our study, even if their median values remained low. The second significant element from our multivariate analysis was the type of RT chosen according to the PET/CT results. Treatment with SBRT (lymph node and/or bone) was negatively associated with BR-FS compared to prostate bed and/or elective pelvic node RT. In addition, it was observed that patients treated with elective pelvic lymph node RT with boost and without ADT more frequently achieved a PSA response at 3–6 months post-RT than those treated with pelvic node SBRT (89.3% vs. 48%). These data concur with De Bleser et al., who suggested that elective pelvic node RT increased node recurrence-FS compared to stereotaxic RT alone in a retrospective study of 506 patients with hormone-sensitive lymph node oligorecurrence (≤ 5 lesions detected, mostly on PET/CT) [45]. It could be interesting to consider the combination of these two prognostic factors with PET/CT imaging data to choose and personalize therapeutic strategies.

Other points seemed to be discussed, such as PSA response assessment at 3–6 months post-RT, which was evaluated in patients who had not received concomitant ADT. It was found that most patients (regardless of the radiopharmaceutical used) obtained a significant reduction in PSA at 3–6 months. Therefore, PET/CT imaging was a reliable reflection of recurrence in some patients. However, particularly for patients with bone localizations, the detection was not complete enough to significantly influence the biomarker value when only image guided SBRT was performed. Previously, Supiot et al. [24] mentioned that innovative PET/CT imaging made it increasingly possible to visualize the “recurrent PC iceberg” tip above the waterline. The authors nevertheless concluded that it remained to be shown that this new knowledge of the disease could allow more effective treatment in relapsing PC by combining systemic therapies and treatments targeting the newly visible disease.

Koerber et al. retrospectively included 86 patients with oligometastatic relapse (≤5 lesions detected by PSMA PET/CT) and treated them with RT ± ADT. They showed higher BR-FS rates at 2 and 3 years (85.1% and 55.1%, respectively) than those in our study [46]. However, their study included a greater proportion of patients receiving concomitant ADT, which may explain this difference. Therefore, adding intermittent ADT to RT seemed to improve the therapeutic response [28,47,48,49,50,51,52]. In another study (OLIGOPELVIS), Supiot et al. showed in 67 patients with oligometastatic pelvic node recurrence (less than 6 lesions seen on FCH PET/CT) that the combination of salvage pelvic RT and intermittent ADT for 6 months seemed to prolong progression-free survival (median to 45.3 months) with limited toxicity [28].

An important point to examine is the toxicity caused by image-guided RT. In our study, early RT tolerance was good, with the occurrence of grade 3 uro-digestive adverse effects in three patients.

Our study had certain limitations, the main one being the retrospective and observational study design, which reduced the level of proof power, but which allowed an initial “routine care” analysis, especially as there was very little data on performances in comparison with the 2 “leadership” radiopharmaceuticals for oligometastatic PC recurrence to guide RT. Let’s analyze the patients in our two groups. We observe that the PET-FCH group patients presented less favorable clinical characteristics than those in the PET-PSMA group, with patients who had more often been treated previously. Moreover, due to the study’s retrospective design, the choice of treatments administered was not controlled (as in a prospective study). The RT schedules could differ according to the different practices in the radio-oncology units. In the absence of sufficient evidence, there are no precise recommendations concerning the targeted treatment of metastases. In this regard, several prospective studies are underway to define the best therapeutic strategy. The OLIGOPELVIS 2 trial (NCT03630666) randomizes patients with pelvic node oligometastatic recurrence (≤5 lesions detected by PSMA or FCH PET/CT) into two groups: one receiving intermittent ADT for 6 months associated with elective RT on pelvic nodes and the other intermittent ADT alone. The PRESTO—GETUG 36 study (NCT04115007) evaluates the efficacy of stereotaxic RT administered on all macroscopic tumour sites (up to 5 metastatic sites diagnosed by PSMA or FCH PET/CT or whole-body MRI) associated with reference treatment in patients with hormone-sensitive CP.

## 5. Conclusions

Targeted RT guided by PSMA PET/CT seemed to improve BR-FS compared to FCH PET/CT in patients with local or oligometastatic recurrence with a low PSA value. Nevertheless, prospective studies are needed to determine whether targeted early treatment with innovative PSMA PET/CT lesion mapping improves long-term clinical outcomes in patients with recurrent PC. 

## Figures and Tables

**Table 1:** Patient characteristics.

	TEP-FCH (N = 53)	TEP-PSMA (N = 70)	*p*-Value
Age at RP (years) Mean (SD) Min; Max	62.6 (6.8) 49; 78	63.2 (6.2) 48; 73	0642
PSA at diagnosis (ng/mL) Mean (SD) Min; Max Missing data	12.37 (24.57) 3.8; 181.0 0	9.60 (6.79) 3.4; 44.0 4	0.381
Initial pT status (%) 2 3 4 Missing data	19 (35.8) 32 (60.4) 2 (3.8) 0	28 (40.6) 41 (59.4) 0 (0.0) 1	0.307
Initial pN status (%) 0 1 x	38 (71.7) 5 (9.4) 10 (18.9)	44 (62.9) 7 (10.0) 19 (27.1)	0.583
ISUP Score (%) 1 2–3 4–5	4 (7.5) 33 (62.3) 16 (30.2)	1 (1.4) 56 (80.0) 13 (18.6)	0.054
R Status (%) R0 R1 Missing data	26 (51.0) 25 (49.0) 2	39 (55.7) 31 (44.3) 0	0.712
Additional treatments before imaging (%) RT prostate bed RT prostate bed + ADT RT prostate bed + elective pelvic nodes RT prostate bed + elective pelvic nodes + ADT ADT alone None	14 (26.4) 12 (22.6) 5 (9.4) 9 (17.0) 1 (1.9) 12 (22.7)	23 (32.9) 12 (17.1) 3 (4.3) 0 (0.0) 1 (1.4) 31 (44.3)	0.552 0.495 0.289 <0.010 0.999 0.014
The rank of recurrence on imaging (%) 1st 2nd–3rd–4th	17 (32.1) 36 (67.9)	35 (50.0) 35 (50.0)	0.065
PSA before imaging (ng/mL) Mean (SD) Min; Max	1.23 (0.55) 0.12; 2.0	0.64 (0.46) 0.15; 1.9	<0.001
PSA DT before imaging (months) Mean (SD) Min; Max Missing data	8.04 (6.97) 0.9; 35.0 2	9.44 (6.69) 1.8; 28.8 0	0.268
EAU Risk Group at BR ^1^ (%) EAU low-risk EAU high-risk Missing data	11 (21.6) 40 (78.4) 2	19 (27.1) 51 (72.9) 0	0.529

^1^ As EAU 2020 recommandations [1]. Abbréviations: RP radical prostatectomy, SD standard deviation, PSA Prostate Specific Antigen, RT Radiotherapy, ADT Androgen Deprivation therapy, PSA DT PSA Doubling Time, BR biochemical recurrence.

**Table 2:** Lesions characteristics detected by PET/CT.

	TEP-FCH (N = 53)	TEP-PSMA (N = 70)	*p*-Value
Number of lesions per patient (%) 1 2 3 4 5	25 (47.2) 19 (35.8) 7 (13.2) 2 (3.8) 0 (0.0)	42 (60.0) 19 (27.1) 5 (7.2) 3 (4.3) 1 (1.4)	0.199
Prostate bed relapse (%) no yes	39 (73.6) 14 (26.4)	53 (75.7) 17 (24.3)	0.836
Pelvic lymph node lesion (%) 0 1 >1	22 (41.5) 19 (35.8) 12 (22.7)	28 (40.0) 22 (31.4) 20 (28.6)	0.737
Extrapelvic lymph node lesion (%) 0 ≥1	44 (83.0) 9 (17.0)	63 (90.0) 7 (10.0)	0.288
Bone lesion (%) 0 ≥1	38 (71.7) 15 (28.3)	60 (85.7) 10 (14.3)	0.071
Visceral lesion (%) 0 ≥1	52 (98.1) 1 ^1^ (1.9)	70 (100.0) 0 (0.0)	0.431

^1^ Cerebral lesion.

**Table 3:** Treatment characteristics guided by PET/CT imaging.

	TEP-FCH (N = 53)	TEP-PSMA (N = 70)	*p*-Value
Radiotherapy (%) Prostate bed only (+/− boost) Elective pelvic nodes + boost Elective para-aortic nodes + boost Prostate bed SBRT Nodes SBRT Bone SBRT Nodes and bone SBRT Visceral SBRT	3 (5.7) 9 (17.0) 1 (1.9) 4 (7.5) 21 (39.6) 8 (15.1) 6 (11.3) 1 (1.9)	11 (15.7) 30 (42.9) 1 (1.4) 1 (1.4) 17 (24.3) 9 (12.9) 1 (1.4) 0 (0.0)	0.082 0.002 0.999 0.164 0.068 0.722 0.042 0.431
ADT concomitant intermittent (%) No Yes <6 months >6 months	30 (56.6) 18 (34.0) 10 8	55 (78.5) 13 (18.6) 12 1	0.028
Other systemic treatment concomitant ^1^ (%)	5 (9.4)	2 (2.9)	0.139

^1^ Durvalumab. Abbreviation: SBRT Stereotactic Body Radiotherapy.

**Table 4:** PSA response at 3–6 months post-RT.

Response Criteria ^1^	TEP-FCH (N = 37) ^2^	TEP-PSMA (N = 57) ^2^	*p*-Value
Complete response (%) (PSA decreased by at least 50%)	17 (46.0)	27 (47.4)	0.607
Partial response (%) (PSA decreased from 10% to 50%)	5 (13.5)	13 (22.8)	
Stability (%) (PSA moved by ±10%)	5 (13.5)	5 (8.8)	
Progression (%) (PSA increased by more than 10%)	10 (27.0)	12 (21.0)	

^1^ As defined in several studies [34,35,36]. ^2^ Only patients who had not received concomitant ADT.

**Table 5:** PSA response at 3–6 months post-RT in relation to the treatment received.

RT Type (%)	PSA Response ^1^ (N = 62)	No PSA Response (N = 32)	*p*-Value
Prostate bed (*n* = 13)	12 (92.3)	1 (7.7)	0.054
Elective pelvic and extrapelvic nodes (*n* = 28)	26 (92.9)	2 (7.1)	<0.001
SBRT pelvic nodes (*n* = 23)	11 (47.8)	12 (52.2)	0.044
SBRT extrapelvic nodes (*n* = 10)	5 (50.0)	5 (50.0)	0.300
Bone SBRT (*n* = 13)	5 (38.5)	8 (61.5)	0.031
Bone and nodes SBRT (*n* = 6)	2 (33.3)	4 (66.7)	0.175
Visceral SBRT (*n* = 1)	1 (100.0)	0 (0.0)	0.999

^1^ Complete and partial responses included (PSA decrease > 10%).

**Table 6:** Uni- and multivariate analyses of predictors of biochemical relapse after treatment.

	HR (95% CI)	Univariate *p*-Value	HR (95% CI)	Multivariate *p*-Value	
Age at diagnosis	1.01 (0.98–1.04)	0.509	-	-	
PSA at diagnosis	1.01 (1.00–1.02)	0.122	-	-	
pT 3–4 vs. 2	1.23 (0.78–1.92)	0.369	-	-	
pN 1 vs. 0	1.21 (0.60–2.45)	0.600	-	-	
ISUP 2–3 vs. 1 4–5 vs. 1	1.05 (0.33–3.37) 1.50 (0.45–5.03)	0.933 0.511	- -	- -	
R Status R1 vs. R0	0.95 (0.62–1.46)	0.815	-	-	
The rank of recurrence on imaging 2–3-4 vs. 1	2.16 (1.34–3.49)	0.002	0.73 (0.41–1.31)	0.290	
RT before imaging Yes vs. No	2.67 (1.61–4.45)	<0.001	1.90 (0.82–4.39)	0.135	
RT type before imaging RT prostate bed vs. None RT prostate bed + pelvic nodes vs. None	2.36 (1.39–3.99) 5.69 (2.82–11.48)	0.001 <0.001	- -	- -	
ADT before imaging Yes vs. No	2.19 (1.40–3.45)	0.001	1.55 (0.92–2.60)	0.097	
PSA before imaging	1.70 (1.18–2.44)	0.004	1.42 (0.85–2.37)	0.178	
PSA DT before imaging	0.96 (0.92–0.99)	0.018	0.94 (0.91–0.98)	0.005	
EAU Risk Group at BR High-risk vs. Low risk	1.90 (1.10–3.28)	0.022	-	-	
Group PET-PSMA vs. PET-FCH	0.54 (0.35–0.85)	0.008	0.97 (0.56–1.70)	0.924	
Lesion number on PET/CT 2 or + vs. 1	1.22 (0.80–1.87)	0.358	-	-	
Lesion localization Pelvic node vs. Prostate bed Extrapelvic node vs. prostate bed Bone/visceral vs. prostate bed	2.68 (1.20–5.97) 6.32 (2.51–15.95) 5.29 (2.25–12.45)	0.016 <0.001 <0.001	1.75 (0.73–4.19) 2.04 (0.70–5.97) 1.25 (0.43–3.68)	0.206 0.194 0.681	
RT Type Elective pelvic node vs. prostate bed Elective para-aortic node vs. prostate bed Node SBRT vs. prostate bed Node and bone SBRT vs. prostate bed Bone SBRT vs. prostate bed	1.37 (0.58–3.23) 4.10 (0.84–19.96) 5.14 (2.26–11.73) 4.65 (1.61–13.40) 4.09 (1.94–11.88)	0.478 0.080 <0.001 0.004 0.001	- - - - -	- - - - -	
Summary of RT type Bed prostate and/or elective pelvic node vs. SBRT	0.32 (0.17–0.60)	<0.001	0.33 (0.18–0.61)	0.001	

Abbreviations: HR Hazard Ratio, CI Confidence Interval, RT radiotherapy, PSA DT PSA Doubling Time, BR Biochemical Recurrence.

## Data Availability

The datasets used and/or analyzed during the current study are available from the corresponding author on request.

## Supplementary Materials

The following supporting information can be downloaded at: https://www.mdpi.com/article/10.3390/cancers15061898/s1. Table S1: Review of the study literature evaluating the clinical impact of PSMA or FCH PET/CT-directed radiotherapy.

## Abbreviations

ADT	androgen deprivation treatment
ADT-FS	androgen deprivation treatment-free survival
BR	biological recurrence
BR-FS	biological recurrence-free survival
EAU	European Association of Urology
FCH	fluorocholine
FCH-PET	[18F]F-choline PET/CT
MDT	metastasis-directed therapies
PC	prostate cancer
PSA	prostate-specific antigen
PSA DT	PSA doubling time
PSMA	prostate-specific membrane antigen
PSMA-PET	[68Ga]Ga-PSMA PET/CT
RP	radical prostatectomy
RT	external radiotherapy
SBRT	stereotactic body radiotherapy
