# Supplementary material for: Comparison of Hormone-Sensitive Oligorecurrent Prostate Cancer Patients Based on Routine Use of Choline and/or PSMA PET/CT to Guide Metastasis-Directed Therapy
Source: Cancers (Basel). 2023 Mar 22;15(6):1898. doi: 10.3390/cancers15061898 (PMC10047404; doi:10.3390/cancers15061898)
Supplement: Supplementary file 1 [file cancers-15-01898-s001.zip › cancers-2228095-supplementary.pdf]

Table S1. Review of the study literature evaluating the clinical impact of PSMA or FCH PET/CT-directed radiotherapy.

**Authors** *(Only Retrospective study)*	**Patients number** ***(lesions)***	**Disease Status**	**Median PSA before PET/CT (ng/mL)**	**Treatment received**	**Median follow up**	**Results**
**Mazzola et al.** [31] **2021**	88 *(118)*	Oligo-metastatic recurrence (≤3 lesions)	PSMA = 0.58 FCH = 2.04	Only SBRT without ADT	25 months	Longer ADT-FS in patients treated with SBRT guided by PSMA vs FCH → ADT-FS at 1 and 2 years: PSMA = 87.8% and 82.3% vs FCH = 73.2% and 63.8% (p=0.010).
**Schmidt-Hegemann et Al.** [33] **2020**	272 *(NA)*	Persistent PSA after RP or 1st BR	PSMA = 0.58 FCH = 0.6	Prostate bed RT ; Elective pelvic nodes RT ± boost ; Metastatic RT ; ± ADT associated *(combination of several RT types)*	30 months	No difference on BR-FS between PSMA or FCH RT guided → BR-FS at 1 and 2 years : PSMA = 63% and 55% vs FCH=73% and 63% (p=0.197). In multivariate analysis, ∀ radiopharmaceutical used →High PSA level before PET/CT was a pejorative factor for BR-FS (p=0.024).
**Deijen et Al.** [32] **2021**	50 *(72)*	Oligo-metastatic only (≤4 lesions)	PSMA = 1.8 FCH = 4.2	SBRT ± ADT associated	24.3 months	Longer ADT-FS if SBRT guided by PSMA vs FCH → Median ADT-FS PSMA = 32.7 months vs FCH = 14.9 months (p=0.010). In multivariate analysis, ∀ radiopharmaceutical used →High PSA level before PET/CT was a pejorative factor for ADT-FS (p=0.025).
**Our Study 2022**	123 *(204)*	Bed prostate or oligo-metastatic recurrence (≤5 lesions)	PSMA = 0.48 FCH = 1.27	Prostate bed RT ± boost ; Elective node RT ± boost ; SBRT ; ± ADT associated *(combination of several RT types)*	42.2 months	Longer BR-FS if RT guided by PSMA vs FCH → median BR-FS PSMA = 24.7 months vs FCH = 13.0 months (p=0.008). Longer ADT-FS if RT guided by PSMA vs FCH → ADT-FS at 2 and 3 years PSMA = 75.9% et 65.5% vs FCH = 49.8% et 24.7% (p=0.001). In multivariate analysis, ∀ radiopharmaceutical used → short PSA TD (p=0.005) and SBRT (p=0.001) were pejorative factors for BR-FS.
